# Beyond awareness: mental health promotion requires epistemic diversity, not pathologization

**DOI:** 10.3389/fpsyt.2026.1775419

**Published:** 2026-05-08

**Authors:** Vojtech Pisl, Vladan Starcevic, Jiri Horacek, Jan Vevera, Timo Beeker

**Affiliations:** 1 Center for Advanced Studies of Brain and Consciousness, National Institute of Mental Health, Klecany, Czechia; 2 Faculty of Medicine in Pilsen, Charles University, Pilsen, Czechia; 3 University of Sydney, Faculty of Medicine and Health, Sydney Medical School, Nepean Clinical School, Sydney, NSW, Australia; 4 Nepean Hospital, Department of Psychiatry, Penrith, NSW, Australia; 5 Third Faculty of Medicine, Charles University, Prague, Czechia; 6 Department of Psychiatry and Psychotherapy, Medical School Brandenburg Theodor Fontane, Neuruppin, Germany; 7 Faculty of Health Sciences Brandenburg, Brandenburg Medical School, Neuruppin, Germany

**Keywords:** resilience, generative AI, mental health awareness, mental health literacy, primary prevention, psychiatrization

## Abstract

Public mental health has become an increasing concern over recent decades, accompanied by a rapid expansion of public awareness campaigns aimed at improving population mental well-being. We argue that while psychiatric interpretations of distress promoted by these campaigns may be beneficial for individuals with clinically significant mental disorders, emerging evidence suggests that the same interpretations may have unintended adverse effects among psychologically healthy individuals. We argue that empirical support for the effectiveness of universal mental health awareness campaigns remains limited and that their benefits are frequently overstated due to selective or overly generalized interpretations of existing studies. We propose that effective mental health promotion should move beyond uniform messaging toward individualized approaches that foster flexibility in how distress is understood and managed. Finally, we suggest that mathematical modelling and artificial intelligence offer promising technical tools for designing such adaptive, individualized mental health promotion strategies, and provide an example of the latter.

## Introduction

Emotions, thoughts, and psychiatric symptoms may spread through mechanisms resembling contagion (1). In clinical domain, such social contagion was demonstrated for mood, anxiety, and eating disorders (2–4), non-suicidal self-injury (5), or suicidal behavior (6). The same social transmission applies to the explanatory frameworks (EF) we use to interpret mental distress and personal hardships as, for instance, a disorder, trauma, or divine punishment (7). These explanations fight relentlessly against each other for space in human limited cognitive capacity that often fails to host more than one explanation. Humans tend to *reify* the explanations of distress they accept, treating hypothetical, reductionist interpretations isolated from our lives as if they explained our mental states exhaustively and absolutely, and they often fail to consider alternatives (8). Thus, reification results in simplified, unambiguous explanations of complex situations, including its construed meaning, causes, and consequences.

The human capacity to cope with threats and adversities depends on the “fit between the strategy and ongoing situational demands” (p. 592) (9). Thus, psychiatric resilience is related to “the ability to utilize a diverse repertoire of regulatory strategies” (p. 605) (9). The resilience research and promotion therefore needs to avoid the fallacy of uniform efficacy: the erroneous presumption that coping mechanisms can be evaluated irrespectively of the contexts of the specific situation and individual (9). Indeed, the PASTOR model of resilience (10) and synthesis of the meaning-making literature (11) emphasize the ability to flexibly assign constructive meanings to adverse situations. Similarly, the growing burden of mental health problems in contemporary society has been linked to a “limited number of rigid interpretation schemes” that complicate the processing of uncertainty (p. 4) (12). Therefore, it can be expected that various explanations of distress may be beneficial to specific individuals in specific situations, just like psychotherapy clients can benefit from various, even contradictory explanations provided by different psychotherapy schools (13).

Even diagnosable psychiatric disorders share, to various degrees, the features of biomedical diseases as well as social constructs (14–16). Consequently, their prevalence is affected by looping effects, because the clinical and scientific classifications of psychopathology shape how individuals express their distress and experience their mental struggles and disorders (17–19). These looping effects are even more important for healthy populations where adopting a specific interpretation cannot be justified by the need for clinical case formulation and treatment. The evolution of various explanations of distress can be seen as a cultural mechanism functioning to “ameliorate anxiety by imbuing the universe with order and meaning, by providing standards of value that are derived from that meaningful conception of reality” (p. 65) (20). Accordingly, it is plausible to conceptualize various EF as meaning-making systems that can foster sense of coherence, regulate affect, structure appraisal, and guide coping responses (11, 21). Therefore, in attempts to form the cultural interpretation of distress in order to improve public mental health, these EF need to be evaluated by their pragmatic benefit for distressed individuals.

Inspired by Brinkman’s classification of languages of suffering (22), we distinguish five EF that ground the causes of distress in different domains. Biomedical and sociopolitical EF interpret our struggles as the result of disturbances in our bodies and the society, respectively (for instance, as illness or injustice). Although they may seem very different at first glance, they both provide an external cause of distress that may reduce the sense of personal responsibility while providing an opportunity to seek medical treatment or to engage in a social cause, respectively. The spiritual EF presents our struggles as experiences with deeper meaning (e.g., divine ordeal), that makes the suffering more bearable. The existential EF frames our struggles as an inherent part of human life, supporting resilience by implying that we are equipped to endure it. Finally, the moral EF portrays our problems as the result of our actions, implying that individuals can actively participate in changing them. The tentative classification is largely arbitrary, and is included to demonstrate the existence of diverse sources or resilience grounded in diverse interpretations rather than to provide a comprehensive list of perspectives with their potential benefits, risks, and limitations.

Deciding objectively which EF is the correct one for a specific situation is impossible because the preference for a given EF is a choice of the observer. Yet, we are noticing unsubstantiated, unwarranted, and counterproductive advantages the biomedical and the sociopolitical EF are receiving in contemporary Western society via mental health literacy (MHL) campaigns. Unsubstantiated, because there is little evidence to support these two EF at the expense of the others. Unwarranted, insofar as its justification is grounded in logically inconsistent and misleading argumentation (23, 24). Counterproductive, because the domination of only two EF deprives people of unique sources of resilience grounded in alternative perspectives.

In the following paragraphs, we first comment on why the benefits of the biomedical and sociopolitical frameworks may be overestimated and how excessive promotion of these EF by MHL campaigns may undermine public mental health. Second, we argue that the effectiveness of such campaigns may be overestimated due to an insufficient distinction between facts and values within the MHL paradigm. Finally, we suggest that individualized MHL campaigns may promote resilience and wellbeing by endorsing a variety of EF, utilizing mathematical modelling or artificial intelligence (AI).

## Explanatory frameworks in traditional and contemporary psychiatry

Psychiatry was developed to treat a minority with severe mental health problems with very low levels of functioning. For instance, the estimates of the prevalence of mental disorders rose from 0.5% in the 1900s to 2% in the 1950s in Sweden (25), while 2% of the US population was considered mentally ill in a sophisticated census in 1880 (26). To treat this minority, psychiatry explained its suffering using predominantly the biomedical and sociopolitical EF: as disorders and traumas. The dissemination of these two EF provided relief from societal expectations, mitigated stigma and self-stigma and provided opportunity for new forms of help, whether pharmacological, psychosocial, or other. The remarkable ability of these explanations to help specific subpopulations in specific contexts has fostered the notion that promoting these two EF to the broad public of the 21st century would also be beneficial. There are at least three reasons making this assumption unjustified.

First, many tasks of today’s psychiatry concern different individuals. Authoritative institutions claim that about one in six is mentally ill in Europe (27) and nearly one in four in the US (28), and growing emphasis on prevention requires psychiatry to increasingly engage with healthy populations, extending its reach to the majority of society. Simultaneously, the concepts of mental health problems have broadened to include less severe and completely novel unpleasant or maladaptive experiences (29, 30). The concept of trauma has evolved from a reaction to extreme environmental stress to an umbrella term for any adversity including mild or moderate distress linked to previous negative experiences (24, 31, 32). These changes have introduced psychiatry to novel, more functional and less disabled individuals, whereby explanations based on medical disorders and trauma may be less effective for more functioning individuals. Moreover, the adoption of the biomedical and sociopolitical perspectives may interfere with the resilience of those who cope with the demands of their lives successfully, without seeking help from mental health services. Moreover, the contemporary focus on the recognition of milder problems as disorders decreases public attention to the more severe mental problems (33) and exhausts clinical capacities needed to help those who cannot be effectively helped in non-clinical contexts (34).

Second, psychiatry increasingly affects society as a whole (17, 34). Applying biomedical and sociopolitical EF to everyday experiences triggers complex changes of cultural narratives. Examples of these developments may include a long-term shift from internal to external locus of control (35). People may be less willing to rely on their inner strength and resilience when encountering difficult emotions, due to the tendency of Western societies to interpret an increasing variety of undesired experiences as mental health matters (29, 31, 36–41). Potential negative effects of such shifts may negate the positive effects the biomedical and sociopolitical explanations may have on individuals with mental disorders. Specifically, the biomedical and sociopolitical EF present suffering as lying beyond individuals’ control, either due to abnormalities of their brains or traumas caused by past injustices, thus undermining one’s sense of agency and diminishing a sense of control. Furthermore, these explanations complicate the acceptance of distress by implying that mental discomfort is abnormal, require attention and expert solution (42). Such suggestions heighten the perceived salience of even minor discomfort. This is reflected in terms like the medicalization of ordinary life] (43), diagnostic cultures (44), or the therapy culture (45).

Third, the public endorsement of biomedical and sociopolitical EF has changed. The positive effects of widespread promotion of biomedical explanations, beneficial in the 20^th^ century, may be limited to societies where the problem of underdiagnosis and undertreatment is substantially larger than the problem of overdiagnosis and overtreatment (46). Yet, in the Western societies, this was no longer the case already a decade ago (47). According to the prevalence inflation hypothesis (19), promotional campaigns encouraging the public to adopt the psychiatric lens trigger a self-perpetuating spiral. Provided with psychiatric explanations, the public has been paying more attention to their mental struggles, feeling less mentally healthy and demanding more treatment and further preventive campaigns. Similarly, promoting sociopolitical EF might only be beneficial when sociopolitical explanations are neglected. But that may no longer be the case today, as the notion that an individual is harmed by the society is steadily becoming more popular (24, 29–31). Consequently, humans may come to be perceived as primarily vulnerable rather than resilient in the face of life’s perils, fostering a cultural transmission of fear (48) and the development of culture of victimhood, where the society rewards weakness rather than strength (49). It can be argued that because sociopolitical phenomena like inequality or violence predict mental disorders, sociopolitical explanations should be promoted as a form of primary prevention (50–53). Yet, it can be also argued that interpreting distress as a result of sociopolitical factors that are not controlled by the individual also has downsides for the individual (24, 48, 54). For instance, while perceived control accelerates recovery from stress (55), extensive notions of harm (56) and blaming the world (57) predict posttraumatic distress. In any specific situation, the positive effects of emphasizing the role of social factors may or may not overweigh the negative implications of the sociopolitical framing for the individual. Thus, interpreting distress as the result of societal factors is not automatically beneficial for mental health.

While the biomedical and sociopolitical explanations may improve recognition of disorders or traumas and reduce stigma, they may also promote self-fulfilling negative expectations (29, 58). Therefore, the question arises about the impact of different EF on public mental health in the contemporary societies. Specifically: Is generic, non-individualized promotion of biomedical and sociopolitical EF through mental health awareness campaigns still justified in the Western society as a strategy of mental health prevention?

## The blind spots of the MHL paradigm

These questions do not receive satisfactory attention, because public mental health campaigns are often only evaluated from the narrow perspective of the MHL paradigm. Under this paradigm, the effectiveness of interventions is presumed to be demonstrated by increases in the scores on various MHL scales. These MHL scores are, however, unrelated to mental wellbeing, according to a meta-analysis (59). Indeed, the necessary evidence to ascertain whether “awareness campaigns” are generally effective, non-effective, or even harmful for public mental health is lacking (60). There are at least three reasons that make it difficult to challenge the effectiveness of awareness campaigns under the MHL paradigm.

First, the meaning of MHL is often obscured and its assessment has not been adequate. MHL was introduced mimicking health literacy, which was defined as the ability to use information to “obtain and maintain good health” (61). Yet, the study introducing MHL already departed from such concept, defining MHL as the “knowledge and beliefs about mental disorders which aid their recognition, management or prevention” (61). Such definition excludes beliefs beneficial to mental health that are unrelated to disorders and includes beliefs that may be detrimental to mental health. For instance, the belief that an individual suffers from depression or ADHD and therefore needs to seek medical help may aid in the recognition and management of disorders (whether or not the individual fits the diagnostic criteria), but may discourage him or her from efficient coping and promote prognostic pessimism. MHL was subsequently defined as a combination of four factors: “understanding how to obtain and maintain positive mental health; understanding mental disorders and their treatments; decreasing stigma related to mental disorders; and, enhancing help-seeking efficacy” (62). The scales measuring MHL, however, depart from such definition, focusing mostly on the knowledge about disorders, often completely omitting an understanding of how to obtain and maintain positive mental health (62–64), and reducing help-seeking to few predefined options, mainly related to entering the mental healthcare system. Consequently, MHL scales may suggest that public campaigns improve MHL, irrespective of whether and how they affect mental health. For instance, celebrities’ social media disclosures of mental disorders have been described both as dangerous for glamorizing mental illness (65) and as “anti-stigma initiatives” that should be encouraged (66). By focusing solely on stigma, potentially iatrogenic interventions may be mistakenly regarded as beneficial.

Second, the uncertainty about whether MHL campaigns improve public mental health is often invisible in reviews of their effectiveness. By focusing on vaguely operationalized MHL instead of a specific outcome, heterogeneous outcomes can be analyzed together, making the resulting average effect unrelated to public mental health or any other specific outcome. On the one hand, studies often report no effect of MHL campaigns on variables measuring mental health or wellbeing (67, 68). On the other hand, many other outcomes are often statistically significant, such as the number of phone calls on a hotline being advertised, increased knowledge of the information promoted by a campaign or increased willingness to seek more information about mental health problems after encouraging the public to seek such information (69, 70). Such results are certainly necessary for campaigns to be effective, but they do not constitute evidence for benefits for public mental health. By diluting findings of little or no effect on mental health with statistically significant, yet largely irrelevant results, reviews generally present the MHL paradigm as successful.

There are several examples of the neglect of the relevant findings or their masking with those that are irrelevant for mental health. A recent review (69) claims that it “highlights … the need for more targeted campaigns” (p. 13), although the review reported no evidence about effects of the campaigns on mental health or wellbeing. Another recent review (70) claims that its results “demonstrate the potential of utilizing media campaigns as a tool for mental health promotion” (p. 918). However, only 3 of 18 included studies reported outcomes related to mental health. Crucially, two of these three trials found no difference between the intervention and control groups, while the third only found a significant effect on a secondary outcome measure (see **Supplementary Materials 1** for overview of the results of the studies included in the reviews). Similarly, a recent meta-analysis titled The Effect of Digital Mental Health Literacy Interventions on Mental Health claims to find a moderate effect of digital mental health literacy interventions in “enhancing distal mental health outcomes” (71). This finding is based on studies about the effects of heterogeneous interventions for diverse populations on mental health outcomes (for instance, an online therapeutical program for patients with psychotic disorders (72) or internet-based cognitive behavioral therapy for patients with rheumatoid arthritis (73)). The effectiveness of these interventions, however, does not imply that increasing MHL (that is often not even mentioned by studies included in the meta-analysis) is related to better mental health, particularly not in the general population.

Third, the lack of evidence regarding the benefits of MHL campaigns for mental health is often unclear due to inadequate adherence to the fact–value distinction. By referring to “improvements” or “positive findings” instead of describing factual results, MHL studies often present ready-made judgements. Both reviews (69, 70) repeatedly claim to report evidence of “improvements” or “positive changes,” despite failing to make even a conceptual distinction between change and improvement. Any effect observed in studies included in the reviews was automatically interpreted as beneficial, without assessing its benefits and costs, or even specifying what should constitute a benefit (see the **Supplementary Materials 1** for excerpts of the studies demonstrating the lacking distinction between an improvement and a change). Thus, the MHL paradigm interprets any manipulation of public attitudes to mental health as an improvement, making it virtually impossible to doubt the alleged success of the campaigns.

In brief, MHL campaigns are often implemented with a persuading power to convince the public to adopt the biomedical and sociopolitical EF, despite a lack of evidence of the benefit of such endeavors. Of course, there are many more complex resilience endeavours other than the awareness campaigns promoting the biomedical and sociopolitical EF, such as those grounded in mindfulness training (74), cognitive-behavioral therapy (75), or experiential learning (76). Yet, most programs known to us are grounded in a single EF that is implicitly assumed to be universally applicable and useful, rather than offering a heterogeneity of perspectives. A notable exception are campaigns grounded in the dialectical behavioral therapy, which explicitly emphasize the tension between acceptance and change (77–79).

## Discussion

### Implications

There are two key implications for the campaigns aiming to improve public mental health. First, the campaigns should prioritize their primary objective of promoting mental health, resilience, and well-being, rather than relying on changes in intermediate or proxy variables, such as specific MHL factors while omitting others. Second, protecting public mental health may require individualized promotion of heterogeneous EF, rather than guiding the public towards the biomedical and sociopolitical explanations only. Thus, we propose that promoting flexible meaning-making and heterogeneity of perspectives facilitating different coping mechanisms may be substantially more beneficial to public mental health than campaigns promoting the biomedical and sociopolitical EF. This proposal should be empirically examined by developing and testing individualized interventions that:

- Empower individuals to feel capable of living the lives they have been thrown into without fostering excessive concerns about their mental health.
- Promote resilience by facilitating adoption of the overlooked or underutilized EF that might be most beneficial in a specific situation.

The suggested interventions supporting diversity of interpretations and coping mechanisms should then be compared to the existing MHL interventions using mental health and wellbeing, rather than knowledge or willingness to seek medical help, as outcome measures.

### Interactive simulations as potential MHL tools

Individualized campaigns that promote a diversity of solutions may require a more complex technological infrastructure than one-size-fits-all educational content. Digital media are used for this purpose, such as interventions targeting multiple variables related to resilience via “therapeutic videogame” (80), attempts to facilitate social-emotional learning in an online game (81), or other (82). Indeed, interactive and experiential MHL campaigns that target specific groups tend to be more effective than those presenting general didactic contents (75, 76). Similarly, campaigns promoting diversity of interpretations might be based on fictional advisors or interactive scenarios, using, for instance, generative AI^1^, or interactive simulations derived from the system dynamics. System dynamics is a mathematical approach to model complex, nonlinear systems using feedback loops. Building on the network models of mental disorders, system dynamics may provide the grounds for interactive simulations of mental struggles and wellbeing (for a feasibility study related to burnout, see (84)). The resulting models of fictional clients can provide the user with various interventions to try to meet the mental needs of the simulated person. By doing so, the users can learn from their own successes and failures in the simulations rather than by passively consuming standardized educational materials. Instead of receiving advice that a particular situation requires a specific solution, users can experiment with various actions linked to multiple EF and learn from subsequent changes in the mental state of a simulated client. Similar simulations have been used to enhance understanding of political decision-making and promote participation in public life (85) or to illustrate the dynamics of the COVID-19 pandemic and explain the rationale behind restrictive and protective measures (86)^2^. As an additional benefit, such approach would promote the grasp of mental health problems as complex phenomena, counteracting the erroneous but widespread public notion of mental disorders as well-defined diseases with sharp boundaries and uniform etiologies. Alternatively, interactive educational scenarios may be based on fictional characters represented by the generative AI, which is already utilized in individualized mental health interventions such as depression and anxiety reduction by facilitating expressive writing (87), or providing insights from “virtual therapists” (88, 89).

Similar strategies can be used to provide users with individualized feedback on their attitudes towards their own struggles. For instance, a digital tool may simultaneously provide feedback from the perspectives of multiple AI-generated personas embodying distinct EF. Such personas can be instantiated through targeted prompting of a generative AI model or via retrieval-augmented generation (RAG), which grounds the epistemic background of each persona in a curated collections of text (e.g., psychiatric literature for a biomedical EF, spiritual texts for a spiritual EF etc). Each persona can thus be understood as an automated application of a specific EF to a specific subjective situation experienced by a specific individual. By combining multiple personas, a single tool can offer diverse, potentially conflicting interpretations, thereby fostering epistemic diversity and flexibility in meaning-making and reappraisal. Moreover, the use of multiple personas and perspectives may mitigate concerns associated with “virtual therapists,” such as biased outputs, illusory therapeutic bonds, or excessive concerns about one’s mental health and self-diagnosing (89–92). To examine this option, we have generated letters from five personas representing the five EF commenting on a life story of a fictional individual or a theatre play script describing a disputation of a committee of these personas (see **Supplementary Materials 2**). The heterogeneous interpretations of specific experiences described in the CV warrant research of potential risks and benefits of providing such individualized heterogeneous feedback to the public.

### Summary

This article argues that while public mental health may benefit from various explanations of mental struggles, many awareness campaigns for the public often promote only the biomedical and sociopolitical EF. The consequent excessive use of these two EF may have adverse effects on public mental health. The perils of such campaigns may be overlooked because of the historical success of these explanations in different contexts and misleading presentation of the results of MHL research. We specifically discuss the possibility that current awareness campaigns are inefficient because they are not tailored to the needs of individuals and provide the same biomedical and sociopolitical contents to all, encouraging people to assume patient and victim roles. Therefore, we propose that (1) the effects of MHL campaigns on mental health and wellbeing needs to be empirically tested, and (2) novel MHL interventions need to be developed that promote the ability to flexibly choose among various conceptualizations of emotional distress. We suggest that system dynamic models and generative AI may provide technical tools for individualized campaigns that promote resilience and wellbeing instead of guiding the public towards specific interpretations of mental distress and personal hardships. Testing the effects of such tools on mental health, wellbeing, and resilience is needed to confirm or disprove our assumption that supporting heterogeneity of interpretations and coping mechanisms may be more beneficial to public mental health than generic promotion of biomedical and sociopolitical explanations.

## Data Availability

The original contributions presented in the study are included in the article/**Supplementary Material**. Further inquiries can be directed to the corresponding author.

## Footnotes

1 “Generative AI – such as ChatGPT – is a type of AI that produces human-like text by predicting the most likely continuation of language based on patterns learned from vast data and can do so according to user instructions and from different perspectives as requested” (83). The previous sentence was generated by ChatGPT as an example of its functioning, in response to a request to describe generative AI in one sentence for an academic article about mental health.

2 For intuitive understanding of such solutions, we encourage the reader to examine the educational simulation of COVID-19 restrictive measures in the CoronaGame (available at koronahra.cz).
