# Supplementary material for: Beyond awareness: mental health promotion requires epistemic diversity, not pathologization
Source: Front Psychiatry. 2026 May 8;17:1775419. doi: 10.3389/fpsyt.2026.1775419 (PMC13195013; doi:10.3389/fpsyt.2026.1775419)
Supplement: Supplementary file 1 [file Table1.docx]

**Beyond awareness: mental health promotion requires epistemic diversity, not pathologization**

**Supplementary Materials 1: illustrations of lack of fact-value distinction in the MHL research**

The lack of rationale for labelling the effects of mental health awareness campaigns as positive is demonstrated on two recent reviews. For each of the reviews, we include three short sections:

1. “eligibility criteria” – in this section, we demonstrate the discrepancy between (1) the eligibility criteria that do not specify particular criteria for what is considered “an improvement” or “positive change”, respectively, and (2) the interpretation of the results that clearly presents the findings as “positive” or “an improvement”, respectively.
2. “presentation versus MH-related outcomes – in this section, we demonstrate that while the effects of awareness campaigns are repeatedly presented as positive, few or none of the studies included in the reviews demonstrated their positive effects of mental health of their consumers.
3. “list and evaluation of the studies” – here we present a list of the studies analysed in the reviews, along with an information (1) whether such a study measured an effect of an awareness campaign on mental health of its consumers, and (2) whether the effect was found to be positive and statistically significant. We would like to emphasize that none of the reviews claims that it was evaluating an effect of the campaigns on mental health.

## Plackett et al 2025

<https://www.jmir.org/2025/1/e68124/>

### Improvement criterion in the eligibility criteria

**Eligibility criteria**

*“the aim of the campaign was to raise awareness of mental health, change attitudes toward mental health, reduce stigma, and encourage help-seeking. This includes mass media campaigns that use other media, but only if there are data on the impact of the social media element.”*

**Description of the aims**

*“This review aimed to assess the effectiveness of social media campaigns in high-income countries in improving knowledge and attitudes toward mental health, reducing stigma, promoting help-seeking behavior, and reaching underserved communities.”*

### Presentation versus MH-related outcomes

**Excerpts from the abstract:**

*attitudes showed the most improvement before and after the campaigns (5/11, 45%), behavior change showed the least positive change over time (1/8, 13%)*

*The evidence highlights the potential of social media campaigns in improving mental health knowledge, attitudes, stigma, and behavior change. However, due to the methodological limitations of these evaluations, it is challenging to determine if the positive changes in these outcomes are a result of the campaigns or other factors.*

**Excerpts from discussion & conclusions of the paper**

*This scoping review underscores the potential of social media campaigns in improving knowledge, attitudes toward mental health, and stigma, and promoting help-seeking behavior. It highlights the potential importance of campaign awareness in contributing to behavior change and the need for more targeted campaigns to reach underserved communities, who may benefit from better mental health information, access to resources, and a focus on empowerment.*

*attitudes toward mental health were found to improve most post campaign compared to precampaign*

*attitudes generally improved before and after campaigns*

**Number of studies included in the review**

**26**

**Number of studies evaluating the effect of the campaigns on mental health**

**0**

**Number of studies finding positive effect**

**NA**

**Number of studies finding no or negative effect**

NA

### List and evaluation of the studies

| **study** | **Study included a statistical test examining the effect of the campaign on participants' mental health** | **Was the result positive and significant?** |
| --- | --- | --- |
| Alvarado-Torres et al (2023) [52] | No (cross-sectional comparison of self-selected samples) | NA |
| Booth et al (2018) [24] | No (help seeking examined) | NA |
| Collins et al (2018) [41] | No (cross-sectional comparison of self-selected samples) | NA |
| Collins et al (2019) [57] | No (cross-sectional comparison of self-selected samples) | NA |
| Collins et al (2020) [42] | No (cross-sectional comparison of self-selected samples) | NA |
| Collins et al (2022) [43] | No (cross-sectional comparison of self-selected samples) | NA |
| Collins et al (2022) [44] | No (cross-sectional comparison of self-selected samples) | NA |
| Collins et al (2022) [45] | No (cross-sectional comparison of self-selected samples) | NA |
| Coughlan et al (2021) [59] | No (no relevant evidence presented) | NA |
| Diouf et al (2022) [51] | No (cross-sectional comparison of self-selected samples) | NA |
| Drane et al (2022) [46] | No (no relevant evidence presented) | NA |
| Evans-Lacko et al (2013) [35] | No (other aspects of MHL examined) | NA |
| González-Sanguino et al (2019) [39] | No (other aspects of MHL examined) | NA |
| Hahn et al (2023) [54] | No (cross-sectional comparison of self-selected samples) | NA |
| Hann and Hemming (2016) [53] | No (no relevant evidence presented) | NA |
| Hansson et al (2016) [55] | No (other aspects of MHL examined) | NA |
| Henderson et al (2016) [36] | No (other aspects of MHL examined) | NA |
| Henderson et al (2017) [37] | No (cross-sectional comparison of self-selected samples) | NA |
| Henderson et al (2020) [40] | No (other aspects of MHL examined) | NA |
| Livingston et al (2013) [48] | No (other aspects of MHL examined) | NA |
| Livingston et al (2014) [49] | No (other aspects of MHL examined) | NA |
| Public Goods Project (2019) [50] | No (Unless the observed increase in the prevalence of self-assessed mental health problems after the campaign is interpreted as a sign of deterioration of mental health) | NA |
| Sampogna et al (2017) [38] | No (other aspects of MHL examined) | NA |
| Santini et al (2022) [47] | No (no relevant evidence presented) | NA |
| Thompson et al (2021) [58] | No (Unless the increased positive response to question “People like me can have a problem with their MH” after the campaign is interpreted as a sign of negative self-evaluation of mental health) | NA |
| Zenone et al (2020) [56] | No (no relevant evidence presented) | NA |

## Tam et al 2024

<https://journals.sagepub.com/doi/pdf/10.1177/15248399241232646>

### Missing positivity criterion in the eligibility criteria

**Eligibility criteria**

*“To be included, we required that studies evaluated an existing media mental health campaign … Campaigns had to focus on mental health issues surrounding emotional disorders such as depression and anxiety, as these are reported by the WHO to commonly impact young people, as well as suicide and selfharm, as it is the fourth leading cause of death among older adolescents (aged 15–19 years). ... Our working definition of a mental health awareness media campaign (hereon “mental health campaign”) is a marketing effort to raise public awareness of mental health issues using an organized set of communication tactics through media platforms including the internet, broadcasting, and print media, and intending to reach a large number of people in the public and generate specific outcomes.”*

**Description of the topic**

*“The campaigns were generally associated with positive changes in the attitudes, beliefs, and intentions of young people (e.g., reduced stigma) and positive changes in behaviors (e.g., increased help-seeking behaviors).”*

### Presentation versus MH-related outcomes

**Excerpt from conclusion in the abstract:**

*The campaigns were generally associated with positive changes in the attitudes, beliefs, and intentions of young people (e.g., reduced stigma) and positive changes in behaviors (e.g., increased help-seeking behaviors)*

**Excerpts from discussion & conclusions of the paper**

*Increase in positive attitudes and awareness and decrease in stigma around mental health are among some of the reported proximal impacts. Intermediate impacts included increase in help-seeking behaviors. The results demonstrate the potential of utilizing media campaigns as a tool for mental health promotion, particularly for a youth demographic group, but also underscore the importance of ongoing, rigorous evaluations of these campaigns to clearly characterize their short- and long-term effects.*

*The present review reports positive effects of mass media campaigns on individuals’ awareness, beliefs, and behaviors around mental health. These findings support the benefits of using mass media to disseminate mental health messages to reach specific groups*

**Number of studies included in the review**

**18**

**Number of studies evaluating the effect of the campaigns on mental health**

**3**

**Number of studies finding positive effect**

**1**

**Number of studies finding no or negative effect**

**2**

### List and evaluation of the studies

| **Study** | **Study included a statistical test examining the effect of the campaign on participants' mental health** | **Was the result positive and significant?** |
| --- | --- | --- |
| [Booth et al. (2018)](https://journals.sagepub.com/doi/full/10.1177/15248399241232646#bibr5-15248399241232646) | No (help seeking examined) | NA |
| [Carli (2016)](https://journals.sagepub.com/doi/full/10.1177/15248399241232646#bibr6-15248399241232646) | Yes | No (a change in the desired direction was found in all participants but there was no significant difference between the treatment and control group. The review, however, cites this study as one of two papers that "found direct effects on individuals in the intervention groups") |
| Cheng et al. (2020) | No | No |
| [Choi et al. (2016)](https://journals.sagepub.com/doi/full/10.1177/15248399241232646#bibr8-15248399241232646) | No (anecdotic qualitative results) | No |
| Cote et al. (2021) | Yes | No |
| [Craig Rushing et al. (2021)](https://journals.sagepub.com/doi/full/10.1177/15248399241232646#bibr11-15248399241232646) | Yes | No (a change in the desired direction was found in all participants but there was no significant difference between the treatment and control group. The review, however, cites this study as one of two papers that "found direct effects on individuals in the intervention groups") |
| [Halsall et al. (2019)](https://journals.sagepub.com/doi/full/10.1177/15248399241232646#bibr17-15248399241232646) | No (help seeking and subjective beliefs about mental-health related knowledge examined) | NA |
| [Jenner et al. (2010)](https://journals.sagepub.com/doi/full/10.1177/15248399241232646#bibr18-15248399241232646) | No (help seeking examined) | NA |
| [Kirchner et al. (2020)](https://journals.sagepub.com/doi/full/10.1177/15248399241232646#bibr19-15248399241232646) | No (anecdotic qualitative results) | NA |
| [La Sala et al. (2021)](https://journals.sagepub.com/doi/full/10.1177/15248399241232646#bibr21-15248399241232646) | No (other aspects of MHL examined) | NA |
| [Livingston et al. (2013)](https://journals.sagepub.com/doi/full/10.1177/15248399241232646#bibr24-15248399241232646) | No (other aspects of MHL examined) | NA |
| [Livingston et al. (2014)](https://journals.sagepub.com/doi/full/10.1177/15248399241232646#bibr23-15248399241232646) | No (other aspects of MHL examined) | NA |
| [McTernan et al. (2023)](https://journals.sagepub.com/doi/full/10.1177/15248399241232646#bibr26-15248399241232646) | No (cross-sectional comparison of self-selected samples of individuals who did or did not choose to watch a documentary in television) | NA |
| [Niederkrotenthaler & Till (2020)](https://journals.sagepub.com/doi/full/10.1177/15248399241232646#bibr28-15248399241232646) | Yes | Partly (RCT finding reduction in depressed mood following exposure to a movie with suicidal topic, while the primary result of the study, expected reduction in suicidal ideation, was insignificant.) |
| [Spears et al. (2016)](https://journals.sagepub.com/doi/full/10.1177/15248399241232646#bibr32-15248399241232646) | No (no relevant evidence presented) | NA |
| [Thompson et al. (2021)](https://journals.sagepub.com/doi/full/10.1177/15248399241232646#bibr34-15248399241232646) | No (Unless the increased positive response to question “People like me can have a problem with their MH” after the campaign is interpreted as a sign of negative self-evaluation of mental health) | NA |
| [Wrobel et al. (2022)](https://journals.sagepub.com/doi/full/10.1177/15248399241232646#bibr38-15248399241232646) | No (no relevant evidence presented) | NA |
| [Zenone et al. (2020)](https://journals.sagepub.com/doi/full/10.1177/15248399241232646#bibr39-15248399241232646) | No (no relevant evidence presented) | NA |
