# Supplementary material for: Beyond awareness: mental health promotion requires epistemic diversity, not pathologization
Source: Front Psychiatry. 2026 May 8;17:1775419. doi: 10.3389/fpsyt.2026.1775419 (PMC13195013; doi:10.3389/fpsyt.2026.1775419)
Supplement: Supplementary file 2 [file Table2.docx]

**Beyond awareness: mental health promotion requires epistemic diversity, not pathologization**

**Supplementary Materials 2: illustrations of individualized feedback from the perspectives of 5 voices inspired by the Brinkman’s (2014) *languages of suffering* created by the generative AI**

Five letters were requested from the ChatGPT (GPT-5.2 by OpenAI), analyzing a narrative CV originally written for a psychotherapy training purposes from the perspective of the five EF, providing insights about the self-evaluations in the CV, as well as advice for sources of resilience the individual can additionally benefit from. Depending on the exact wording of the prompts, the letters provided diverse perspectives along with kind support, confrontations, and advisory. Furthermore, the chatbot was requested to provide discussions that these five personas may hold about the individual, framed as a theatrical disputation or as a fictional committee meeting.

## Letters: example of letters from personifications of different explanatory frameworks generated by GPT-5

### 1. Biomedical Voice – requesting confrontational tone

*“Dear Jake — the man with symptoms”*

Dear Jake,

You are a brilliant diagnostician of others, but you forget that you too are a patient. I read your story and want to grab your shoulders and say: stop calling your melancholy “temperament.” It’s not temperament — it’s persistent dysthymia with anxious features. You’ve normalized your insomnia, your hypervigilance, your lifelong unease, as though they were quirks of an intelligent man. No. They are signs of chronic activation, a system too long exposed to conflict and uncertainty.

When you say you “like doing mind,” I hear compulsive activation — an avoidance pattern reinforced by positive feedback from achievement. You think you are choosing; I say your amygdala is choosing for you.

You mention your grandmother — heroic, resilient, dragging wood on children’s sleds. But you glorify her *symptoms*: overcompensation, denial of frailty, self-sacrifice. You learned endurance, not regulation. That legacy still burns your nervous system.

Think about your sleepless childhood nights not as metaphors of solitude, but as unprocessed hyperarousal. Think about your “low stress tolerance” as neurological fatigue, not a flaw of will. Seek treatment, not meaning. Stop giving poetic explanations to biological exhaustion.

You are wrong when you think you are free from illness. You are one of us. Welcome.

With clinical concern,
Dr. Diagnosticus

### 2. Spiritual Voice – requesting short advise on subclinical sleep problems

*“Dear Jake — the one who turns prayer into planning”*

Dear Jake,

You call it insomnia, but I call it the hour when your soul refuses anesthesia. The dark is not your enemy; it’s the moment heaven tries to speak and you keep refreshing your mind like an inbox.

You inherited your grandmother’s stubborn grace, but not her surrender. She could sit with silence; you must conquer it. That’s why you lie awake.

What to do?
Stop treating night as a failed day.
When you can’t sleep, pray — not for sleep, but for stillness. Let the racing thoughts become psalms. Whisper gratitude instead of hypotheses.
You’re not broken, Jake. You’re being called.

With a candle for your night,
Sister Sophia

### 3. Existential Voice – requesting more individualization

*“Dear Jake — the man who mistook motion for meaning”*

Jake,

You’ve been running since you were fourteen — through call centers, universities, countries, relationships. You call it exploration; I call it avoidance of despair. Each new start was a way to delay the moment when you’d have to ask the terrible question: *What if none of this will ever feel enough?*

You thought stability would cure it — the new house, the academic success, the relationship that “might be” something. But even now, you write with the tone of a man who has built a home and still feels transient. You have not yet learned to dwell.

Stop explaining your anxiety as family heritage or neurotransmitter imbalance. Those are defenses against what you truly fear: that existence is groundless, that meaning must be chosen, not discovered.

Think differently, Jake:

- Your sleepless nights aren’t malfunctions; they’re moments when life refuses anesthesia.
- Your fear of commitment isn’t neurosis; it’s your terror of finitude — that to choose one life means losing all others.
- Your melancholy isn’t weakness; it’s the exact measure of your freedom.

You must stop waiting for peace. There is none. There is only the courage to stand awake.

Yours in shared absurdity,
The Existential Friend

### 4. Moral Voice – requesting confrontational tone

*“Dear Jake — the one who mistakes reflection for righteousness”*

Dear Jake,

You are proud of your self-awareness, and rightly so. But tell me, what good is your insight if it never leaves the room of your own mind? You dissect your parents’ flaws with surgical precision, then absolve yourself through empathy. That is not forgiveness. That is intellectual mercy, the coward’s virtue.

You speak of “boundaries” and “protecting your joy.” Convenient words. Sometimes they are simply shields against your own failure to love. You pity your mother’s dependence, yet you mirror her isolation. You hide behind professionalism — “I help people” — while remaining emotionally stingy in the places where it matters most.

You write warmly of Havel and Cohen, yet you imitate their melancholy, not their responsibility. Havel risked prison; you risk emotional discomfort. Jake, you are too polite to be good.

Think about your hesitation to care for your mother not as “protecting your joy,” but as withholding compassion out of fear. Think about your reluctance to commit in relationships not as prudence, but as ethical cowardice disguised as self-knowledge.

You are wrong if you think decency is natural. It is an act of will. Do the right thing even when it scrapes your pride.

With stern affection,
The Moral Witness

### 5. Sociopolitical Voice– requesting short advise on subclinical sleep problems

*“Dear Jake — the sleepless worker of late capitalism”*

Dear Jake,

You think you can’t sleep because of childhood or temperament. Wrong. You can’t sleep because the system trained your nervous system to monetize every hour. You internalized productivity so deeply that even rest feels unproductive — even dreams must justify themselves.

Your body’s tired, but your ideology won’t let it rest. You are the perfect neoliberal subject: exhausted and still “doing mind.”

What to do?
Treat sleep as resistance.
Refuse screens, clocks, notifications — the technologies of vigilance.
When you lie down, say: *“I’m not available.”*
Your insomnia isn’t a disorder, Jake; it’s the rebellion that forgot it was rebellion.

In solidarity,
The Sociopolitical Voice

## Theatre play: two versions of excerpts provided by GPT-5

### 🌑 Option 1: Theatrical / Philosophical Dialogue

*Title: “The Five Languages Sit in Judgment”*
*(A darkened room. Five figures seated around a wooden table. Above them, the ceiling drips time. A file labeled “Jake” lies open. Each speaks in their own rhythm and light.)*

BIOMEDICAL *(with the precision of a scalpel)*:
Let’s begin with the obvious: persistent dysthymia, mild anxiety, perhaps an avoidant personality structure. The boy never learned downregulation — not in the cradle, not in the lab. Look at the sleeplessness, the overfunctioning, the chronic overidentification with competence. Classic symptom cluster.

MORAL *(interrupting)*:
Symptoms? You reduce his longing to neurotransmitters again! What you call “avoidant,” I call “reluctant to love.” Jake’s insomnia isn’t a disorder; it’s guilt that has forgotten its name.

EXISTENTIAL *(leaning forward)*:
No, it’s not guilt, it’s fear. He stares into the dark because it’s the only time the abyss stares back. You all want to cure him; I want him to stay awake. That’s where freedom begins.

SPIRITUAL *(quiet, resonant voice)*:
Freedom? His sleeplessness is not freedom — it’s prayer in disguise. You keep calling his ache pathology or courage, but it’s a soul’s hunger for the sacred. He listens to the silence but refuses to name it God.

SOCIOPOLITICAL *(slamming a hand on the table)*:
God! Abyss! Disorder! You’re all hypnotized by your inner worlds. The man is the product of history. The divorced parents, the anxious ambition, the lonely intellectualism — these aren’t metaphysics. They’re the offspring of class tension and neoliberal self-exploitation. His sleeplessness is capitalism in the body!

BIOMEDICAL *(coldly amused)*:
Ah, so now cortisol levels are capitalism’s fault? How poetic. No, comrade. He needs medication and rest, not Marx.

SOCIOPOLITICAL *(snarling)*:
You medicalize oppression. You call social alienation “maladaptation.” That’s violence with a clinical smile.

SPIRITUAL *(gently)*:
And you politicize mystery. Don’t you see? All these categories are shadows around a sacred wound. What he needs is surrender — a kind of yielding neither of you can measure.

EXISTENTIAL *(raising a brow)*:
Surrender? To what? That’s precisely his problem — he keeps surrendering to explanations, never to experience. He hides behind frameworks because he fears absurdity.

MORAL *(cutting in)*:
Experience is not enough. He’s clever about his wounds, yet stingy in his care. Look how he describes his mother — as a problem to manage, not a person to forgive. He owes her something. He owes the world decency.

BIOMEDICAL *(dryly)*:
He owes himself serotonin.

SOCIOPOLITICAL:
He owes solidarity.

SPIRITUAL:
He owes reverence.

EXISTENTIAL:
He owes awareness.

MORAL:
He owes love.

*(A silence. The file trembles in a small breeze that has no source.)*

SPIRITUAL *(softly)*:
Perhaps we all see only the reflection that flatters our domain.

EXISTENTIAL *(bitter smile)*:
That’s existence — partial, unredeemed.

SOCIOPOLITICAL:
Then at least make the partial useful. Let his private suffering point outward.

MORAL:
And let his outward service point inward, toward conscience.

BIOMEDICAL:
Fine. Treat the body first. Then he may choose whichever poetry suits him.

*(They all sigh — a weary, human noise. The file remains open. No verdict. The lights dim as each language dissolves into its own silence.)*

### 🕯️ Option 2: Committee-Style Transcript

*Excerpt from the “Interdisciplinary Board on the Interpretation of Jake’s Life.”*
*(Meeting held at the Institute for Human Meaning, transcript recorded by a neutral observer.)*

Chair: The committee will now review the case of *Subject: Jake*. The objective is to determine the dominant explanatory framework for his suffering.

Biomedical Representative: Thank you, Chair. To summarize: long-term affective instability, high-functioning anxiety, possible learned hypervigilance. Genetic predisposition likely. Early environmental stressors. Recommend medical and behavioral management.

Moral Representative: I object to the purely medical reading. The patient’s tone indicates an evasion of moral duty. He intellectualizes pain to avoid guilt. His estrangement from his mother is not pathology — it is a failure of compassion.

Biomedical: You call compassion a cure for chronic insomnia?

Moral: I call love the only sustainable regulation of the heart.

Existential Representative: Both of you misread the context. Jake’s insomnia is not a symptom or a sin; it’s an encounter with nothingness. His problem is not illness but cowardice before freedom. He builds order to escape contingency.

Religious Representative: And you all ignore the sacred dimension. He speaks of yearning but offers no prayer. His grandmother is the saint of his story, but he inherits her endurance, not her faith. He must learn to kneel again.

Sociopolitical Representative: Kneeling won’t help. He’s kneeling already — before the gods of self-optimization. The so-called “doing mind” he glorifies is the neoliberal algorithm internalized. His fatigue is social, not existential.

Chair: Point of order — please refrain from ideology labeling.

Sociopolitical: I call it sociology. He’s been taught that self-worth equals productivity. You can’t fix that with medication or mysticism.

Biomedical: You can, however, lower cortisol.

Existential: You can also confront mortality.

Moral: Or confess.

Religious: Or surrender.

Chair: Order, please. The board must formulate provisional conclusions.

*(Murmur of pages. The observer notes an air of mutual irritation, but also fascination.)*

Chair: Very well. Summary findings —

1. The Biomedical member concludes that Jake’s suffering is primarily medical and treatable.
2. The Moral member asserts that suffering persists due to unacknowledged ethical failure.
3. The Existential member defines the suffering as ontological and necessary for freedom.
4. The Religious member interprets suffering as a spiritual invitation neglected by the subject.
5. The Sociopolitical member insists it arises from structural alienation and social injustice.

*(A pause.)*

Chair: Do we have consensus?

*(Five simultaneous “No”s.)*

Chair: Then the case remains open.

*(The recorder notes: “Each member departed convinced that the others were gravely mistaken — and that Jake himself, in his silence, was the true enigma.”)*
